# Decidual Macrophages Are Significantly Increased in Spontaneous Miscarriages and Over-Express FasL: A Potential Role for Macrophages in Trophoblast Apoptosis

**DOI:** 10.3390/ijms13079069

**Published:** 2012-07-20

**Authors:** Sabine Guenther, Thomas Vrekoussis, Sabine Heublein, Birgit Bayer, David Anz, Julia Knabl, Iordanis Navrozoglou, Darius Dian, Klaus Friese, Antonis Makrigiannakis, Udo Jeschke

**Affiliations:** 1 Department of Obstetrics and Gynecology, Innenstadt Campus, Ludwig-Maximilians University of Munich, Munich D-80377, Germany; E-Mails: honeybine@gmx.de (S.G.); thomas_vrekoussis@yahoo.gr (T.V.); sabine.heublein@med.uni-muenchen.de (S.H.); julia.knabl@med.uni-muenchen.de (J.K.); darius.dian@med.uni-muenchen.de (D.D.); klaus.friese@med.uni-muenchen.de (K.F.); 2 Department of Legal Medicine, Ludwig-Maximilians University of Munich, Munich D-80377, Germany; E-Mail: birgit.bayer@med.uni-muenchen.de; 3 Department of Internal Medicine, Pharmacological ward, Ludwig-Maximilians University of Munich, Munich D-80377, Germany; E-Mail: david.anz@med.uni-muenchen.de; 4 Department of Obstetrics and Gynecology, Medical School, University of Ioannina, Ioannina 45110, Greece; E-Mail: inavro@yahoo.gr; 5 Department of Obstetrics and Gynecology, Grosshadern Campus, Ludwig-Maximilians University of Munich, Munich D-80377, Germany; 6 Department of Obstetrics and Gynecology, Medical School, University of Crete, Heraklion 71409, Greece; E-Mail: makrigia@med.uoc.gr

**Keywords:** macrophage, spontaneous miscarriages, FasL

## Abstract

Decidual macrophages (DM) are the second most abundant population in the fetal-maternal interface. Their role has been so far identified as being local immuno-modulators favoring the maternal tolerance to the fetus. Herein we investigated tissue samples from 11 cases of spontaneous miscarriages and from 9 cases of elective terminations of pregnancy. Using immunohistochemistry and dual immunofluorescence we have demonstrated that in spontaneous miscarriages the DM are significantly increased. Additionally, we noted a significant up-regulation of macrophage FasL expression. Our results further support a dual role for DM during pregnancy and miscarriages. We hypothesize that the baseline DM population in normal pregnancy is in line with an M2 phenotype supporting the ongoing gestation. In contrast, during spontaneous miscarriages, the increased FasL-expressing population could be a part of an M1 phenotype participating in Fas/FasL-related apoptosis. Our results highlight a new aspect of macrophage biology in pregnancy physiology and pathophysiology. Further studies with larger samples are needed to verify the current results and evaluate their clinical impact.

## 1. Introduction

The effectiveness of human reproduction is rather limited. About 70% of the spontaneous conceptions will be lost during the first trimester. Most of them (85%) are lost in a pre-clinical phase, prior to any clinical verification of pregnancy; the rest are expected to be lost at a later clinical stage during the first trimester [1]. Several causes have been attributed to miscarriages. Chromosomal abnormalities, although not often verified, are considered a common cause. Other reported causes are intra-uterine infections, endocrinological/immunological disorders, anatomical problems of the uterus and pharmaceutical/chemical effects on embryo implantation and development [2].

The most important site in early pregnancy maintenance is the fetal-maternal interface. Both the embryonic and the maternal parts are in constant molecular crosstalk, a complex process that involves apart from trophoblast and decidua- several cell types mainly of immunological origin. The aim of this process is a closely regulated trophoblast invasion with a concurrent maternal immune tolerance to the semi-allogenic fetus [3]. Several immune cells have been reported as participants in the fetal-maternal interface. Among these, the most-studied are T and uterine Natural killer (NK) cells; several reports have already demonstrated these cell types as crucial in embryo implantation and early development [4,5]. Decidual macrophages (DM) are the second more abundant population in the maternal-fetal interface [6]. They produce a plethora of cytokines mainly as part of their primary role as antigen presenting cells (APC). In early pregnancy they are described as potential regulators of the T-cell activation/activity, especially when the latter is considered harmful for the ongoing pregnancy [7]. However, their actual role in early pregnancy has not been entirely clarified yet.

The maternal fetal tolerance has been attributed among others to the Fas/Fas ligand (FasL) system [3]. FasL is a transmembrane protein that by binding to Fas receptor, triggers apoptosis to the Fas-expressing cells [8]. In that view, trophoblast and decidual FasL-expressing cells induce apoptosis to the Fas-bearing activated T-cells of the maternal-fetal interface, thus minimizing the risk of embryo rejection during implantation and early development [9]. Recently, T-cells have been also reported to express FasL in abortive material, and induce apoptosis to Fas expressing trophoblast cells *in vitro* [10]. With the aim of further clarifying the role of macrophages during early development, we have investigated the macrophage population in cases of spontaneous miscarriages abortion and elective terminations of pregnancy. Knowing already that (a) FasL is important both during implantation and miscarriages; and (b) that DM express FasL [11], we further checked whether macrophage FasL expression was deregulated in case of spontaneous abortions.

## 2. Materials and Methods

### 2.1. Tissue Samples

This study was approved by the Ethical Committee of the Medical School, Ludwig-Maximilian-University of Munich. Informed consent was obtained from each patient.

Samples from elective terminations of pregnancy (ETP, 9 cases) were provided from private practice clinics in Munich, Germany. Maternal and gestational age-matched tissues samples from spontaneous miscarriages (SM, 11 cases) were additionally collected from the archives of the Department of Obstetrics and Gynaecology, Innenstadt, Munich, Germany. The mean age of women in case of spontaneous abortions was 35.5 ± 1.9 years and in elective terminations of pregnancy 35.4 ± 2.2 years (*p* > 0.05). The mean gestational age for the group of the SM was 69.45 ± 2.54 days and for the group of the ETP was 61.66 ± 6.04 days (*p* > 0.05). Samples were obtained by dilatation and evacuation without any prior pharmaceutical induction. In cases of spontaneous abortion, evacuation was performed within the first 24 h after diagnosis.

All women included in the study had a null medical and family history. History taking was systematic, aiming to exclude–apart from common disorders–possible implication of clotting disorders and autoimmune diseases, already known as aggravating factors for increased risk for miscarriages. In all samples caryotypic analysis excluded chromosomal abnormalities. Additionally, in all samples microbiology analysis excluded possible intra-uterine infection. All women had a normal first trimester vaginal swab.

Following evacuation, specimens from both ETP and SM were immediately immersed in formalin and embedded in paraffin or snap frozen and sectioned with a cryostat.

### 2.2. Immunohistochemistry

Formalin-fixed paraffin-embedded sections (3 μm) were deparaffinized in xylol, rehydrated in a descending ethanol gradient and subjected to epitope retrieval in a pressure cooker using sodium citrate buffer (pH 6.0). After returning to room temperature, sections were blocked with 3% H_2_O_2_ in methanol (20 min) for endogenous peroxidase activity. Non-specific binding of the primary antibodies was blocked by using the appropriate blocking solution. Incubation with the primary antibodies followed. Salient features of the antibodies used are presented in Table 1. Reactivity was detected by using the Vectastain Elite ABC-Kit (Vector Laboratories, Burlingame, CA, USA) according to the manufacturer’s protocol. Substrate and chromogen (3,3′-diaminobenzidine DAB; Dako, Glostrup, Denmark) were finally added to the slides, which were then counterstained with Mayer’s acidic hematoxylin and covered. Replacement of the primary antibody with mouse or rabbit IgG served as negative control.

Apoptotic cells (M30 positive cells) were quantified as the percentage of positive cells over the total number of cells counted per field. For a cell to be counted, it was imperative for the nucleus to be distinguished. Accordingly, macrophages (CD68 positive cells) were quantified as the percentage of positive cells over the total number of cells counted per field (400×). All cases were evaluated and scored by two observers. Evaluation was performed by examining three random fields per case/slide. Both observers were blinded regarding the groups of the study.

### 2.3. TUNEL Assay

After completing the M30 immunostaining, frozen sections from three cases per group were assessed for apoptosis by applying the TUNEL assay (Roche, Germany) according to the manufacturer’s protocol. Apoptotic cells were quantified as the percentage of the TUNEL positive nuclei over the total number of nuclei recognized in the field. Three fields were assessed per case/slide.

### 2.4. Double Immunofluorescence

- **FasL/CD68**. Frozen sections were thawed, fixed in acetone for 5 min at room temperature and rehydrated in PBS. After blocking for 15min with blocking solution (Ultra V Block, Lab Vision, Fremont, CA, USA), the sections were incubated with rabbit-Anti-Fas-L (Q20 Santa Cruz Biotechnologies, Santa Cruz, CA, USA) 1:100 overnight at 4 °C and washed with PBS; incubation at room temperature with goat-anti-rabbit Cy-3 (Jackson Dianova, Hamburg, DE) diluted 1:500 for 30 min followed. After washing with PBS, the slides were incubated at room temperature with mouse-anti-CD68 (clone EBM 11, Dako, Hamburg, Germany) diluted 1:100 for 1 h. Incubation with the goat-anti-mouse Cy-2 secondary antibody (Jackson Dianova, Hamburg, Germany) diluted 1:100 for 30 min at room temperature, followed.
- **HLA-G/M30**. Thawing, fixation, rehydration and blocking were done as described above. The slides were then incubated with the biotinylated mouse-Anti-M30 (CytoDeath, clone M30, Enzo Life Sciences, Lörrach, DE, Germany) diluted 1:100 overnight at 4 °C, succeeded by the incubation at room temperature with streptavidin Cy-5 (Jackson Dianova, Hamburg, DE, Germany) diluted 1:400 for 30 min. Finally the slides were incubated with the FITC conjugated mouse-Anti-HLA-G (clone MEM G/9, Serotec, Oxford, UK) diluted 1:50, overnight at 4 °C.

All antibodies were diluted in dilution medium (Dako, Hamburg, Germany) to minimize background staining. The slides were finally embedded in mounting buffer containing 4,6-diamino-2-phenylindole (DAPI, Vectastain, Vector Laboratories, Burlingame, CA, USA) and examined with a Zeiss Axiophot photomicroscope (Jena, Germany). Pictures were taken with a digital camera (Axiocam MRm, Zeiss, Jena, Germany). The number of FasL-expressing macrophages was expressed as a percentage of the FasL(+)/CD68(+) cells over the total number of CD68(+) cells counted in each slide. For better discrimination between the DAPI blue staining and the Cy-5 blue staining, in the HLA-G/M30 double immunofluorescence staining, nuclei were set as white.

### 2.5. Statistical Analysis

Differences between groups were assessed with the Mann-Whitney test. Correlations were assessed by Spearman test. All observations with *p* < 0.05 were considered significant.

## 3. Results

### 3.1. Apoptosis Is Increased in Spontaneous Abortions Compared to Normal Pregnancies

**Figure 1:**
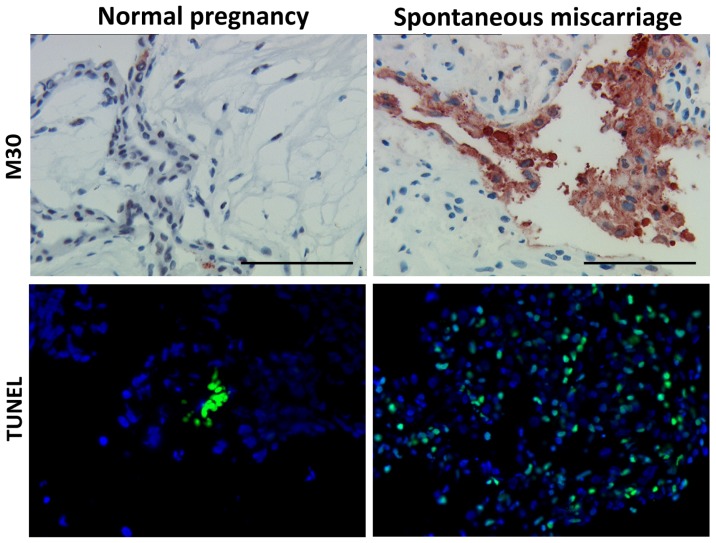
Early pregnancy placental tissue (from either elective terminations of pregnancy or spontaneous miscarriages) stained with anti-M30 cytodeath and TUNEL. It is clear that apoptosis is more intense in cases of spontaneous abortions compared to normal pregnancy. Bar = 100 μm.

**Figure 2:**
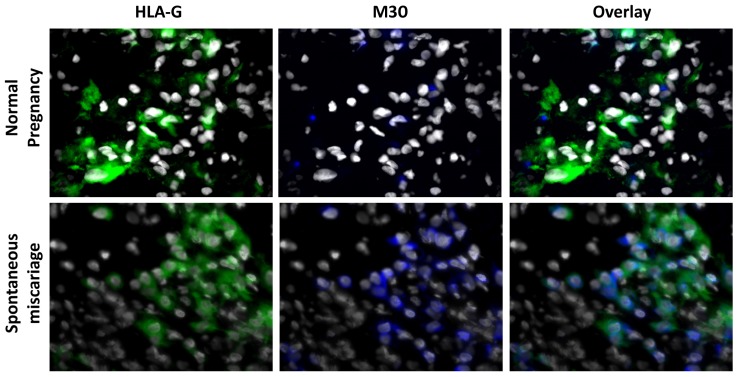
Dual immunofluorescence using HLA-G (an extravillous trophoblast marker) and anti-M30 cytodeath reveals that trophoblast apoptosis is markedly increased in spontaneous abortions.

In order to verify the previously reported induction of apoptosis in spontaneous miscarriages, decidual samples from 11 miscarriage cases and from nine cases of elective pregnancy termination were assessed by immunohistochemistry. The apoptosis marker used in the current study was the well-accepted anti-M30 antibody reactivity. It was found that apoptosis was significantly more abundant in spontaneous abortion tissue compared to normal pregnancies (mean percentage 48.18 ± 4.63% *vs.* 18.88 ± 5.32% respectively, *p* = 0.003) (Figure 1). The TUNEL assay verified these results (mean percentage 67.22 ± 11.40% *vs.* 13.48 ± 5.90% respectively, *p* = 0.049) (Figure 1). Having proved that anti-M30 and TUNEL yield equivalent results, it was decided for the next experiment to be performed using the anti-M30 as apoptotic marker. To clarify further the apoptotic scheme on trophoblasts dual immunofluorescence against HLA-G—an extravillous trophoblast cell-specific marker—and anti-M30 was performed. The apoptotic extravillous trophoblast cells were quantified as the percentage of M30(+)/HLA-G(+) cells over the total number of HLA-G(+) cells. It was revealed that there were significantly more apoptotic extravillous trophoblast cells in spontaneous abortions compared to normal pregnancy (71.81 ± 11.02% *vs*. 10.00 ± 2.9% respectively, *p* = 0.001) (Figure 2).

### 3.2. Macrophage Population and Distribution Are Significantly Altered in Spontaneous Miscarriages

**Figure 3:**
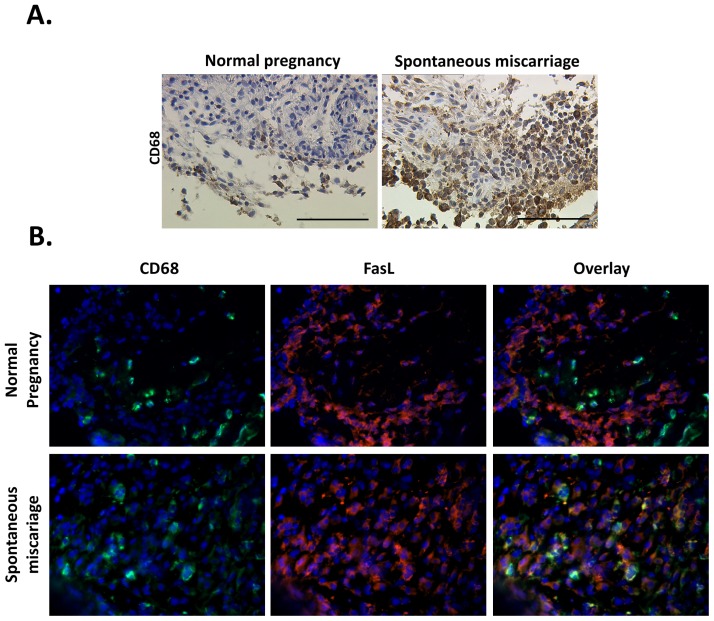
(**A**) Immunohistochemistry against CD68 (a macrophage marker) reveals that in normal pregnancy macrophages are present near the trophoblast. In case of spontaneous abortions the number of macrophages is increased; additionally it seems that they are significantly infiltrating the trophoblast. (**B**) Dual immunofluorescence against CD69 and FasL revealed that the latter’s expression is significantly increased in macrophages involved in spontaneous miscarriages.

Aiming to investigate the existence pattern of the macrophages in SM, both tissue samples from SM and ETP were immuno-stained against the macrophage marker CD68. It was found that macrophage population is significantly increased in spontaneous abortions compared to normal pregnancies (mean percentage 51.36 ± 6.46% *vs.* 27.77 ± 5.95% respectively, *p* = 0.021). Additionally macrophages seemed to infiltrate the decidua in spontaneous abortions, while, in normal pregnancy samples, macrophages were located in the periphery of the trophoblast cells (Figure 3A). The percentage of macrophages per field seems to correlate significantly with the gestational age in the ETP group (Spearman R = 0.75, *p* = 0.01). On the contrary in the SM group this correlation is not significant (Spearman R = −0.21, *p* = 0.53). Despite this significant correlation in the ETP group, the finding about the significant difference in size of the DM population between SM and ETP is still strong. Even though in normal pregnancy the macrophage population seems to increase as gestational age progresses, it does not reach the corresponding population size of the spontaneous miscarriage group; in fact it could be hypothesized that in smaller gestational ages the current difference could be even more significant. A larger sample is needed though to highlight such differences.

### 3.3. Macrophages Significantly Over-Express Fas ligand (FasL) in Spontaneous Miscarriages

Since FasL has already been reported to be involved in SM pathophysiology, it was decided to assess macrophages regarding their possible FasL expression. In that view, both tissue samples from SM and ETP were immuno-stained with double immunofluorescence against CD68 and FasL. FasL-expressing macrophages were quantified as the percentage of FasL(+)/CD68(+) cells over the total number of CD68(+) cells. It was found that in spontaneous abortions the percentage of FasL(+)/CD68(+) cells is significantly higher compared to the corresponding percentage in normal pregnancies (72.72 ± 8.21% *vs*. 45 ± 9.44% respectively, *p* = 0.035) (Figure 3B).

## 4. Discussion

Decidual macrophages (DM) are the second more abundant population identified in the fetal maternal interface [6]. Although less studied during implantation and early pregnancy compared to other immune cells, several actions have been attributed to them. Firstly, they act in an immunosuppressive way regarding a possible maternal immune reaction to the semiallogenic embryo. It has been shown that DM but not peripheral monocytes can inhibit T-cell responses most possibly via prostaglandin E_2_ production [12,13]. Furthermore, DM produce a significant amount of interleukin-10 (IL-10), the latter being recognized as an anti-inflammatory mediator [14]. Simultaneously, DM produce tryptophan metabolites that can effectively suppress T-cell proliferation [15–17]. These actions support a role for DM in contributing to maternal fetal tolerance. It has been shown that DM can produce a wide range of cytokines locally supporting both a pro- and an anti-inflammatory molecular scheme. In analogy to the so described T-cell microenvironment Th1/Th2/T17 polarization [18] macrophages are expected to be polarized as well, recognized as M1 and M2 respectively [19,20]. Thus, the M1 phenotype is related to inflammation as macrophages secrete TNFa and IL-12 [21]. However, M2 phenotype–being anti-inflammatory–is characterized by increased IL-1 receptor antagonist and decreased IL-12 production [7]. Additionally, M2 macrophages express the macrophage mannose receptor (MMR) that mediates host defense and removal of substances produced during inflammatory processes [22,23]. Taking all the above together, it can be easily speculated that an M2 phenotype can be anticipated during normally developing pregnancy. Alternatively, the M1 phenotype could be a part of mechanism leading finally to pregnancy failure. However, it is now accepted that even this approach is rather simplified. Macrophages have been reported presenting dynamic plasticity; their response to external stimuli can vary, ranging from M1 to M2 phenotypes [24]. Thus, it can be anticipated that instead from acting exclusively as of the M1 or M2 phenotypes, macrophage subpopulations can act differently in a particular site and clinical condition. This justifies the fact that despite all the above mentioned functions of DM, their role has not been entirely clarified.

Few reports exist on macrophages regarding population size differences in miscarriages. Vassiliadou and Bulmer had reported an increase was found in macrophage population in case of SM [25]; although the authors considered it as non significant, it is commendable that this increase marginally did not reach significance (*p* = 0.06) [25]. At the same time Quack *et al*., have demonstrated that macrophage populations do not differ in size among samples from ETP and samples from recurrent abortions (with male or female embryo) [26]. Herein, we have shown that during spontaneous miscarriages the DM population increases significantly. This deregulation may possibly support an inflammatory scheme further triggering abortive procedures. Although initially could be considered as contradictory, our results are in partial agreement with the first report since a marginally non-significant increase was noted then [25]. The second report had focused on recurrent miscarriages [26], a pathophysiological entity that is considered rather different than that of spontaneous miscarriages. Of course spontaneous and recurrent miscarriages are expected to share a percentage of pathophysiological mechanisms; nonetheless recurrent miscarriages are considered a rather complicated event involving potentially unknown pathways as well. In that view, a different role for macrophages could be implemented. Taking into consideration at the same time the plasticity by which macrophages could act, both our findings in SM and the previous findings on recurrent miscarriages could hold. Moreover, despite the fact that the previous studies have used bigger samples than the ones used herein, all samples are of comparable size. Vassiliadou and Bulmer had enrolled 20 SM and 21 ETP cases for macrophage evaluation [25], while Quack *et al*., had enrolled 38 recurrent miscarriages and 20 ETP cases [26]. Larger series are to clarify definitely whether macrophage population increases in SM.

Additionally, we have highlighted the existence of FasL in DM both during pregnancy and spontaneous miscarriages. Previously, FasL had been reported as being expressed by monocyte-derived macrophages and that HIV infection could up-regulate FasL intracellular content [27]. Furthermore, macrophages could induce FasL-mediated apoptosis in a mouse model of pulmonary silicosis [28]. In line with these results it was shown that macrophages could induce FasL-mediated apoptosis in Schwann cells, thus leading to chronic demyelinated neuropathy [29]. Our results are in accordance with a previous study demonstrating that DM express FasL [11]. More interestingly, it has been shown herein for the first time that FasL expression in DM is increased during spontaneous miscarriages.

Based on the current findings it can be hypothesized that baseline FasL expression by DM is a part of an M2-like polarization. FasL-expressing DM could induce apoptosis to Fas-bearing activated T-cells reducing potentially harmful immune responses against the semi-allogenic embryo. This hypothesis, if proven correct, could reveal a complementary mechanism of immune tolerance since it has already been shown that decidua and trophoblast cells induce T-cell apoptosis via the Fas/FasL system [9].

The role of FasL-expressing DM in spontaneous miscarriages seems though to be more complicated. Recently, it was shown by our group that, in spontaneous miscarriages, T-cells express FasL achieving a Fas-mediated trophoblast cell apoptosis *in vitro* [10]. This effect was further investigated and was finally attributed to elevated concentrations of the stress-related peptides CRH and Urocortin [10]. In line to our previous findings, FasL-expressing DM could also mediate trophoblast apoptosis highlighting an alternative way of inducing apoptosis in spontaneous miscarriages. Interestingly, peripheral blood mononuclear cells were reported to express CRH receptor 1 [30]. It could be thus speculated in analogy to our previous findings on T-cells, that macrophages over-express FasL as a result of increased CRH concentration. Further *in vivo* and *in vitro* experiments are certainly required to verify this thesis. Alternatively, and in concurrence with the above suggested role of FasL expression by the DM in normal pregnancy, FasL over-expression of macrophages could be considered a counter balance mechanism aiming to reduce the already triggered T-cell mediated apoptosis. Taking into consideration the plasticity with which macrophages act, it could be speculated that both the proposed mechanisms could be active, the one counter-acting the other.

Finally, in the present study it was shown that the apoptotic rate (and especially the trophoblast cell apoptotic rate) was significantly elevated in spontaneous miscarriages. The current finding is in accordance to previous reports in the field [10,31]. Despite the possible role of DM and their FasL expression in regulating apoptosis in normal pregnancy and spontaneous miscarriages, there is always the fundamental question whether the increased apoptosis noted in spontaneous miscarriages is part of the mechanism leading to embryonic death or whether embryonic death is the one triggering apoptosis as a scavenger process against the remains of the trophoblast. In the present study, despite the fact that the evacuation of the abortive material was performed within 24 h from diagnosing the miscarriage, an answer to this crucial question cannot be given. Perhaps this could be achieved by diagnosing the SM the time it occurs and by performing the evacuation of the endometrial cavity immediately after embryonic death. Such approach would ensure that no further apoptosis could interfere with the results. Furthermore, in the current setting it could be implied that, apart from the fact that the exact moment in time the SM happened could not be possibly specified (making impossible to clarify whether apoptosis is the cause or the result of the SM), even this small delay (maximum 24 h) from SM diagnosis to evacuation could affect the extent of apoptosis found. Although this could be true, to the best of our knowledge there is no experimental setting able to overcome this problem as well. In our opinion, the ideal immediate SM diagnosis and thereafter the ideal immediate evacuation after such diagnosis, together consist an experimental approach rather difficult to perform. Firstly, this would demand a daily follow-up with transvaginal ultrasound up to a certain gestational age in the first trimester, a fact that very few pregnant women would tolerate. Moreover, an immediate evacuation after setting the diagnosis of SM is frequently not accepted by the patients, since it is always necessary to immediately overcome the psychological impact of the unpleasant news, and be further informed about the procedure and their future fertility. The potential problems of the operative schedule overload and of the suitability of the patient for immediate anesthesia (pre-operative assessment and the abstinence from meals some hours before anesthesia) further contribute to the delay between SM diagnosis and evacuation. In that view, an evacuation within the first 24 h after SM diagnosis is the best option offered to these patients.

In conclusion, in the present study it was shown that DM population is increased during spontaneous miscarriages. More importantly, it was demonstrated that decidual macrophages’ FasL expression is up-regulated during spontaneous miscarriages. These findings support further the regulatory role of macrophages in early pregnancy physiology and pathophysiology. Due to the small sample size the current findings are considered with caution. Larger samples and properly designed *in vitro* and *in vivo* (animal models) experiments are needed to verify our results and further strengthen the role of DM in reproductive immunology.

## Figures and Tables

**Table 1:** Salient features of the antibodies used in immunocytochemistry.

Antibody (AB)	AB Incubation Conditions	Blocking Solution	Blocking Conditions
mouse-Anti-CD68 (clone EBM 11, Dako, Hamburg, Germany)	1:100, 1, RT	Power block (Dako, Hamburg, DE, Germany)	1:10 in distilled water, 3 min, RT
mouse-Anti-M30 (CytoDeath, clone M30, Enzo Life Sciences, Lörrach, Germany)	1:400, overnight, 4 °C	Ultra V Block (Labvision, Fremont, CA, USA)	undiluted, 45 min, RT

## References

[1] Macklon N.S., Geraedts J.P., Fauser B.C. (2002). Conception to ongoing pregnancy: The “black box” of early pregnancy loss. Hum. Reprod. Update.

[2] Barnea E, Barnea E., Hustin J., Jaunieaux E. (1992). Epidemiology, Etiology of Early Pregnancy Disorders. The First Twelve Weeks of Gestation.

[3] Warning J.C., McCracken S.A., Morris J.M. (2011). A balancing act: Mechanisms by which the fetus avoids rejection by the maternal immune system. Reproduction.

[4] Piccinni M.P. (2010). T cell tolerance towards the fetal allograft. J. Reprod. Immunol.

[5] Yagel S. (2009). The developmental role of natural killer cells at the fetal-maternal interface. Am. J. Obstet. Gynecol.

[6] Houser B.L. (2012). Decidual macrophages and their roles at the maternal-fetal interface. Yale J. Biol. Med.

[7] Nagamatsu T., Schust D.J. (2010). The contribution of macrophages to normal and pathological pregnancies. Am. J. Reprod. Immunol.

[8] Nagata S. (1994). Fas and Fas ligand: A death factor and its receptor. Adv. Immunol.

[9] Makrigiannakis A., Zoumakis E., Kalantaridou S., Coutifaris C., Margioris A.N., Coukos G., Rice K.C., Gravanis A., Chrousos G.P. (2001). Corticotropin-releasing hormone promotes blastocyst implantation and early maternal tolerance. Nat. Immunol.

[10] Minas V., Jeschke U., Kalantaridou S.N., Richter D.U., Reimer T., Mylonas I., Friese K., Makrigiannakis A. (2007). Abortion is associated with increased expression of FasL in decidual leukocytes and apoptosis of extravillous trophoblasts: A role for CRH and urocortin. Mol. Hum. Reprod.

[11] Wongweragiat S., Searle R.F., Bulmer J.N. (2001). Expression of Fas/Fas ligand by decidual leukocytes in hydatidiform mole. Biol. Reprod.

[12] Mizuno M., Aoki K., Kimbara T. (1994). Functions of macrophages in human decidual tissue in early pregnancy. Am. J. Reprod. Immunol.

[13] Parhar R.S., Yagel S., Lala P.K. (1989). PGE2-mediated immunosuppression by first trimester human decidual cells blocks activation of maternal leukocytes in the decidua with potential anti-trophoblast activity. Cell Immunol.

[14] Heikkinen J., Mottonen M., Komi J., Alanen A., Lassila O. (2003). Phenotypic characterization of human decidual macrophages. Clin. Exp. Immunol.

[15] Miwa N., Hayakawa S., Miyazaki S., Myojo S., Sasaki Y., Sakai M., Takikawa O., Saito S. (2005). IDO expression on decidual and peripheral blood dendritic cells and monocytes/macrophages after treatment with CTLA-4 or interferon-gamma increase in normal pregnancy but decrease in spontaneous abortion. Mol. Hum. Reprod.

[16] Munn D.H., Zhou M., Attwood J.T., Bondarev I., Conway S.J., Marshall B., Brown C., Mellor A.L. (1998). Prevention of allogeneic fetal rejection by tryptophan catabolism. Science.

[17] Renaud S.J., Graham C.H. (2008). The role of macrophages in utero-placental interactions during normal and pathological pregnancy. Immunol. Invest.

[18] Saito S., Nakashima A., Shima T., Ito M. (2010). Th1/Th2/Th17 and regulatory T-cell paradigm in pregnancy. Am. J. Reprod. Immunol.

[19] Gordon S. (2003). Alternative activation of macrophages. Nat. Rev. Immunol.

[20] Mills C.D., Kincaid K., Alt J.M., Heilman M.J., Hill A.M. (2000). M-1/M-2 macrophages and the Th1/Th2 paradigm. J. Immunol.

[21] Devaraj S., Jialal I. (2011). C-reactive protein polarizes human macrophages to an M1 phenotype and inhibits transformation to the M2 phenotype. Arterioscler. Thromb. Vasc. Biol.

[22] Chieppa M., Bianchi G., Doni A., Del Prete A., Sironi M., Laskarin G., Monti P., Piemonti L., Biondi A., Mantovani A. (2003). Cross-linking of the mannose receptor on monocyte-derived dendritic cells activates an anti-inflammatory immunosuppressive program. J. Immunol.

[23] Gustafsson C., Mjosberg J., Matussek A., Geffers R., Matthiesen L., Berg G., Sharma S., Buer J., Ernerudh J. (2008). Gene expression profiling of human decidual macrophages: Evidence for immunosuppressive phenotype. PLoS One.

[24] Sica A., Mantovani A. (2012). Macrophage plasticity and polarization: *In vivo* veritas. J. Clin. Invest.

[25] Vassiliadou N., Bulmer J.N. (1996). Immunohistochemical evidence for increased numbers of ‘classic’ CD57+ natural killer cells in the endometrium of women suffering spontaneous early pregnancy loss. Hum. Reprod.

[26] Quack K.C., Vassiliadou N., Pudney J., Anderson D.J., Hill J.A. (2001). Leukocyte activation in the decidua of chromosomally normal and abnormal fetuses from women with recurrent abortion. Hum. Reprod.

[27] Dockrell D.H., Badley A.D., Villacian J.S., Heppelmann C.J., Algeciras A., Ziesmer S., Yagita H., Lynch D.H., Roche P.C., Leibson P.J. (1998). The expression of Fas Ligand by macrophages and its upregulation by human immunodeficiency virus infection. J. Clin. Invest.

[28] Borges V.M., Falcao H., Leite-Junior J.H., Alvim L., Teixeira G.P., Russo M., Nobrega A.F., Lopes M.F., Rocco P.M., Davidson W.F. (2001). Fas ligand triggers pulmonary silicosis. J. Exp. Med.

[29] Dace D.S., Khan A.A., Stark J.L., Kelly J., Cross A.H., Apte R.S. (2009). Interleukin-10 overexpression promotes Fas-ligand-dependent chronic macrophage-mediated demyelinating polyneuropathy. PLoS One.

[30] Angioni S., Petraglia F., Gallinelli A., Cossarizza A., Franceschi C., Muscettola M., Genazzani A.D., Surico N., Genazzani A.R. (1993). Corticotropin-releasing hormone modulates cytokines release in cultured human peripheral blood mononuclear cells. Life Sci.

[31] Pestka A., Toth B., Kuhn C., Hofmann S., Wiest I., Wypior G., Friese K., Jeschke U. (2011). Retinoid X receptor alpha and retinoids are key regulators in apoptosis of trophoblasts of patients with recurrent miscarriages. J. Mol. Endocrinol.

